# Technology enabled non-physician health workers extending telemedicine to rural homes to control hypertension and diabetes (TETRA): A pre-post demonstration project in Telangana, India

**DOI:** 10.1371/journal.pone.0211551

**Published:** 2019-02-19

**Authors:** Shailendra Dandge, Panniyammakal Jeemon, P. S. Reddy

**Affiliations:** 1 Department of Pharmacology, MediCiti Institute of Medical Sciences, Hyderabad, Telangana, India; 2 Society for Health Allied Research & Education, India (SHARE INDIA), Hyderabad, Telangana, India; 3 Sree Chitra Tirunal Institute for Medical Sciences and Technology, Trivandrum, India; 4 University of Pittsburgh, Pittsburgh, PA, United States of America; University of Braunschweig - Institute of Technology, GERMANY

## Abstract

**Objectives:**

We aimed to determine the feasibility and effectiveness of an intervention anchored on mHealth and task sharing strategy of involving non-physician health workers (NPHW) on population level detection, treatment and control of hypertension and diabetes in India.

**Methods:**

Non-physician health workers (NPHWs) equipped with tablet computers that were linked with point-of-care devices for blood pressure (BP) and blood sugar measurements visited households, screened adult individuals for hypertension and diabetes from two randomly selected villages in the Medchal district, Telangana, India. Further, they digitally connected those individuals with hypertension and diabetes to a study physician via Skype, and handed over a printed e-prescription. Medication adherence checks, BP and fasting blood sugar measurements were done once a month and doctor consultations once in three months during follow-up.

**Results:**

Among 2456 eligible individuals, 1751 and 1686 individuals were screened for hypertension and diabetes, respectively. Prevalence of hypertension was 23·6% (95% CI 21·6%-25·6%) and among them 38.9% were newly detected. Prevalence of diabetes was 11·2% (9·7%-12·7%) and 28.6% of them were newly detected. After 24 months of intervention, control of BP and blood sugar was achieved in 54.0% and 34·1% of individuals with hypertension and diabetes, respectively. Blood pressure control rate improved by 12% (7.9%-16.0%) in known hypertensive individuals over the intervention period.

**Interpretation:**

This research demonstrates the feasibility and local acceptability of a mHealth intervention strategy anchored on NPHWs guided by physicians for detection, treatment and regular follow-up of individuals with hypertension and diabetes in a community setting in India.

## Introduction

Currently, 28% of all deaths in India are attributable to cardiovascular diseases (CVD)[1]. Hypertension and diabetes are two major risk factors of CVD. For example, hypertension attributes 48% and 18% of all stroke and Coronary Heart Disease (CHD) deaths in India, respectively[2,3]. Furthermore, individuals with diabetes are reported to be at two to four fold-increased risk of CHD compared to those without diabetes[4]

Hypertension and diabetes awareness, treatment and control are abysmally poor in India[5–7]. Achieving better treatment and control rates of both hypertension and diabetes are crucial to contain the epidemic of CVD in India. In resource poor settings, task-sharing strategy of involving non-physician health care workers (NPHW) has been recognised as a useful strategy for achieving better treatment and control rates of both blood pressure(BP) and blood sugar. Additionally, the mHealth technology has the potential to facilitate the task-sharing strategies by enabling the NPHW with new skills in cardiovascular risk reduction. We conducted a recent national summit of sixty leading cardiovascular researchers in India and recommended technology enabled NPHW in management of both hypertension and diabetes [8].Based on the recommendations of the national summit, we developed a tablet computer based digital health tool to enable NPHWs with a decision support system for management of hypertension and diabetes in community settings. In this paper, we describe the feasibility, and impact of a novel strategy anchored on technology enabled NPHW in management of hypertension and diabetes.

## Materials and methods

### Study settings

The Medchal district in Telangana, India has 40 villages and 10,176 households with a total population of 49,617. SHARE (Society for Health and Allied Research) INDIA developed a GPS (Global positioning system) mapped database called the REACH (Rural Effective Affordable Comprehensive Health Care)database, with a listing of each resident in all the households in the Medchal district by name, date of birth, and gender [9]. We selected two villages from Medchal district by simple random sampling method.

### Study population

Adult male and female participants aged 20 years and above residing in the selected two villages were identified from the REACH database. We approached all the eligible adults residing in the selected two villages for participation in the study. Individuals who were not reachable, not willing to provide consent for participation, pregnant and lactating women were excluded. The study started in July, 2015 and completed in August, 2017.

### Intervention strategies

The key strategies of the intervention were *task sharing* and mHealth. The NPHW worked under the supervision of a physician, and connected to him through a tele-medicine platform as part of the digital health tool (mHealth).

#### Description of the non-physician health workers

We engaged Accredited Social Health Activist (ASHA) as NPHW in the study and trained them in using a customised mHealth tool to identify and manage hypertension and diabetes in community settings. The ASHAs cater to a population of approximately 1000 individuals and are the first point of contact for healthcare needs of the community. They work closely with the Auxiliary Nurse Midwife (ANM) at the health sub-center (the sub-unit of a primary health center, caters to a population of ~5000). The ANMs are a pivotal workforce at the sub-centre level facilitating access to and providing care for maternal and child health services especially those related to antenatal care, immunization, acute respiratory tract infections and diarrhoeal illnesses[10,11].

We deployed three ASHAs—two in one and one in the other village proportional to the size of population and designated them as study NPHWs. Additionally, we deployed two ANMs -one in each village. The ASHAs networked with the ANMs to get blood samples drawn from eligible participants as per the study protocol. The services of the ANMs were required only for 4 months spread over a year. The ANMs deployed in this project had formal training and certification from an accredited institution as per the guidelines of the regulatory authority for nursing education in India.

All NPHWs engaged in the study were provided with intensive in-office training for one week and subsequently for twelve weeks in the field. The training curriculum included the process of household data collection, use of tablet computer, sphygmomanometer, glucometer, printer and arranging Skype interview between the participant and the physician. The NPHWs performed various study related tasks including, questionnaire administration, BP measurement, fasting blood sugar measurement using capillary blood, uploading data on tablet PC, and networking with the study physician on Skype under direct supervision of specially trained supervisors during the twelve weeks field training period. In order to compensate for the time of each NPHW, we paid an honorarium of Rs.3000 per month (equivalent to ~45 US dollars per month).

#### Description of the physician, and supervisor involved in the study

The study physician was a medical doctor with MBBS degree and attended telemedicine calls between 7:30 am and 12:30 pm, six days a week. Additionally, two supervisors-one per village were engaged to train the NPHWs in the field and also to monitor the activities of NPHWs. The supervisors were trained medical social workers with prior community based experience in managing health problems.

#### Description of other project team members

A project manager with primary training in computers and electronics handled technological troubleshoot requests received from the NPHW and the doctor. The project manager also supervised the work of the supervisors and the NPHW on a day-to-day basis.

#### Collection of data

The NPHWs guided by a supervisor visited each household and identified eligible individuals’ for screening. The NPHWs collected information related to medical history, obtained readings of BP and fasting blood sugar. The screening data of all participants were linked to the REACH project database. A structured study questionnaire was administered to gather information on general medical condition, past and present illnesses in local language and responses were noted in a tablet computer. The questionnaire also included sections on self-reported use of tobacco and alcohol, history of medical conditions such as hypertension, diabetes, bronchial asthma, thyroid disease, kidney disease, cardiovascular disease, and neurological diseases. Additionally, name, dose and duration since use of current medications were recorded.

#### The digital health tool (mHealth)

**Fig 1:**
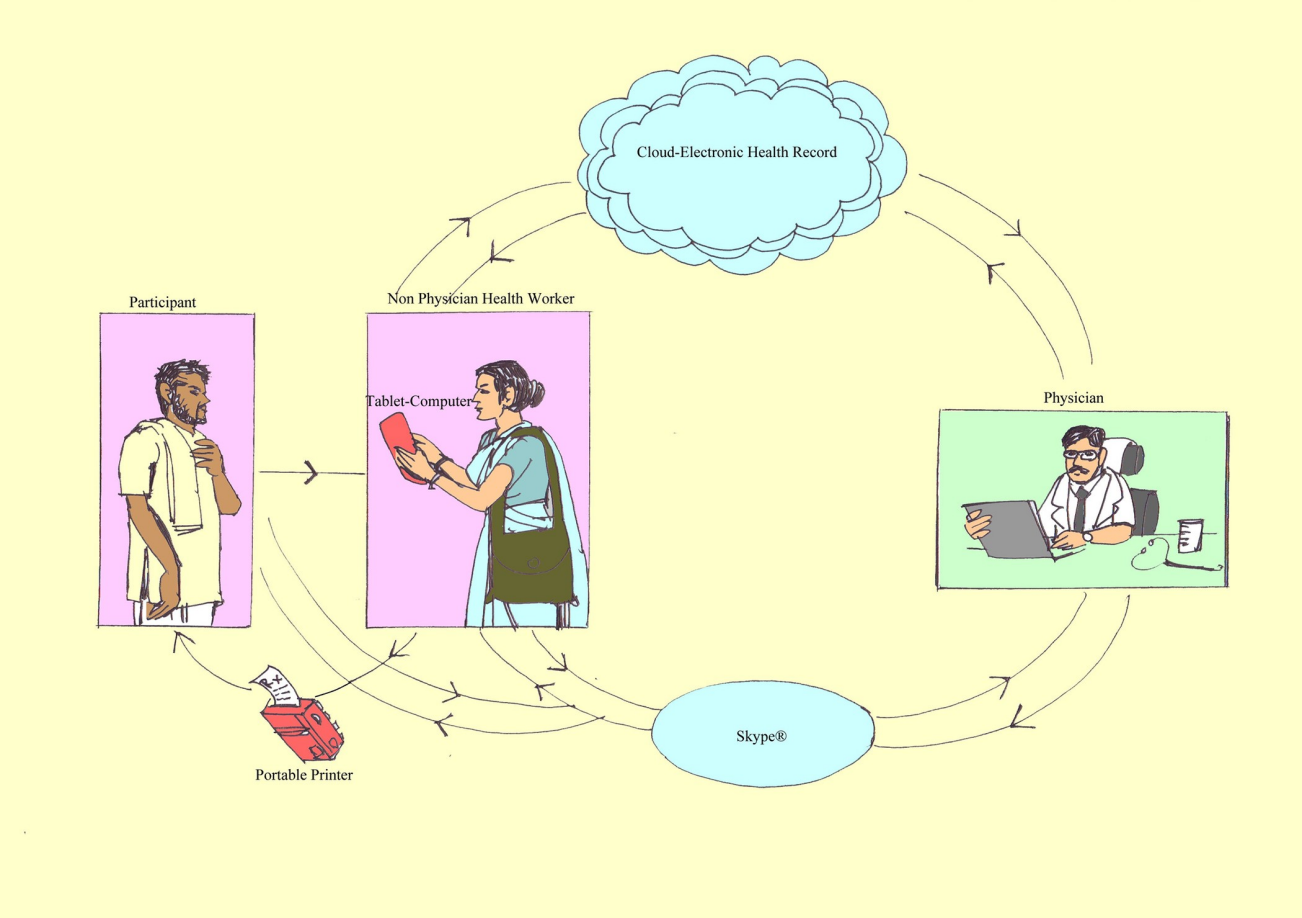
Intervention strategy.

We developed an in-house mHealth application on Windows. The mHealth application was installed on the assigned tablet computers given to the NPHW. The application facilitated easy access to the screening tool, and all the study questionnaires. The mHealth application also generated a daily list of the participants due for follow-up visits and provided alerts to the NPHW to schedule Skype calls with the physician when required. Additionally, it created an electronic health record (EHR) for all study participants. Internet access in all the given tablets enabled the NPHW to synchronize the EHR with a cloud server. The study physician reviewed the EHR directly on his computer dashboard. The study physician also received a real-time alert on his mobile phone for an immediate tele-appointment. The NPHW with the help of the mHealth application facilitated a video Skype call between the study participant and the physician. Based on the tele-medicine consultation with the participant and the details available in the EHR, the study physician recommended appropriate management (Fig 1). The treatment recommendations were recorded on the EHR, uploaded on the cloud server, and accessed by the NPHW in real-time on her tablet computer device. The NPHW printed the recommended treatment by using a wireless printer attached to the tablet computer and the print-out was given to the patient. Routine medicines were supplied for a month in the newly diagnosed cases and they were encouraged to approach either Government or private distribution system for further supply. Based on the treatment recommendations, the study NPHW distributed medicines for one month and scheduled subsequent monthly follow-up visits. The real-time data analytic interface as part of the mHealth tool allowed the project team to monitor the progress of screening, follow-up, treatment and control status. Weekly performance review report was also provided to each study NPHW. The electronic health record was maintained securely with passcode protection with access only to the assigned study team members.

#### Screening for hypertension and diabetes

**Blood pressure measurement and diagnosis of hypertension**

We used Rossmax (model MJ701f) automated sphygmomanometer for BP measurements. The automated sphygmomanometer was interfaced with the tablet computer for automated input of measurements, which were subsequently uploaded on to a secure cloud system via internet. All the BP measurements were integrated with the individual’s medical history, other clinical parameters, and maintained as part of an electronic health record (EHR). The calibration protocol for the automated sphygmomanometer was followed as per the manufactures instruction manual. In addition, the automated BP sphygmomanometers were cross checked once a month with a manual mercury sphygmomanometer by a trained professional. In case there was a difference of more than 10 mm Hg in systolic or 5 mm Hg in diastolic between the two devices, the automated sphygmomanometer was discontinued from use and sent for calibration.

Three BP readings were obtained from all study participants on the right arm using an appropriate sized cuff, with the patient comfortably seated, and arm at the level of the heart. All readings were obtained with at least one minute gap between two consecutive readings. The first systolic and diastolic BP readings were ignored, and an average of the second and third readings was considered as the systolic and diastolic BP for that occasion. The first blood pressure reading was disregarded to improve the accuracy of BP readings based on the theoretical possibility that the first reading of blood pressure may be elevated as compared to the subsequent measurements.

Hypertension was diagnosed if the first visit systolic BP was ≥180 or diastolic BP ≥110 mm Hg in those without past medical history of hypertension. Among others without a past medical history of hypertension, follow up measurements of two more sets of three BP measurements were obtained on two different days within the first week of recruitment. Hypertension was diagnosed if the average of 2^nd^ and 3^rd^ systolic BP measurements across the three visits was ≥140 mm Hg or the average of 2^nd^ and 3^rd^ diastolic BP across the three visits was ≥90 mmHg. All those who were on anti-hypertensive drugs were included in the hypertension category irrespective of their BP levels.

**Blood sugar measurement and diagnosis of diabetes mellitus**

Capillary Blood sugar was measured with Codefree glucometer. The glucometer was interfaced with the tablet computer and the data were automatically gathered as part of EHR. Diabetes mellitus was suspected if fasting blood sugar (FBS) was ≥126 mg/dl. Among individuals with suspected diabetes, venous blood was drawn by the study ANM at the participant’s household and was transported to an accredited central laboratory. Glycated haemoglobin (HbA1c) test was performed by high performance liquid chromatography. An individual with FBS ≥ 126mg/dland HbA1c ≥ 6.5% was diagnosed to have diabetes. All those on anti-diabetic drugs were also included in the diabetes category irrespective of their blood sugar levels.

**Serum creatinine**

Serum creatinine estimation was done for all the subjects diagnosed with hypertension and /or diabetes. Serum creatinine estimation was done by Jaffe’s Kinetic method. The HbA1c and serum creatinine results were directly integrated with the EHR system from the laboratory itself. Serum creatinine was used by the study physician for decision making while prescribing metformin and antihypertensive drugs.

**Blood pressure and blood sugar control definitions**

The intended duration of follow-up was two years. Assessment of blood sugar was done monthly during the follow-up and HbA1c at endline. Blood sugar control was defined as HbA1c<7% or FBS<126mg/dl and analysis presented according to both criteria. Blood pressure control was defined as systolic BP<140 mm Hg and diastolic BP <90 mmHg.

### Data analysis

Data were summarised as mean± SD for continuous variables and proportions for categorical variables. Comparison of paired proportions (before and after the intervention) was done by McNemar’s chi-square test. As post-hoc analyses, a new variable on overall control status was defined that summarised the control status at all follow-up visits. Adequate control of blood sugar and BP during follow-up visits was defined as achievement of fasting blood sugar <126 mg/dl and BP <140/90 mm Hg respectively in more than 60% of the follow-up visits. Additionally, mean levels of BP, blood sugar and HbA1c (paired comparisons between baseline and endline) were compared using paired t test.

As an indicator for operational feasibility and the performance of NPHW, the percentage of individuals with hypertension and/or diabetes successfully followed-up from the listing of those due for monthly and quarterly follow-up was assessed. The data were analysed using STATA version 13.

### Protection of the human subjects

We explained the objectives, methods, benefits, and risk of our study to the participants and took written informed consent. We used unique identification numbers for each participant to mask their identity and maintain confidentiality. Institutional Review Board of MediCiti Institute of Medical Sciences, Hyderabad, India approved the study. Confidentiality of EHR was ensured by providing passcode-protected access only to authorized study personnel.

## Results

### Baseline characteristics

**Fig 2:**
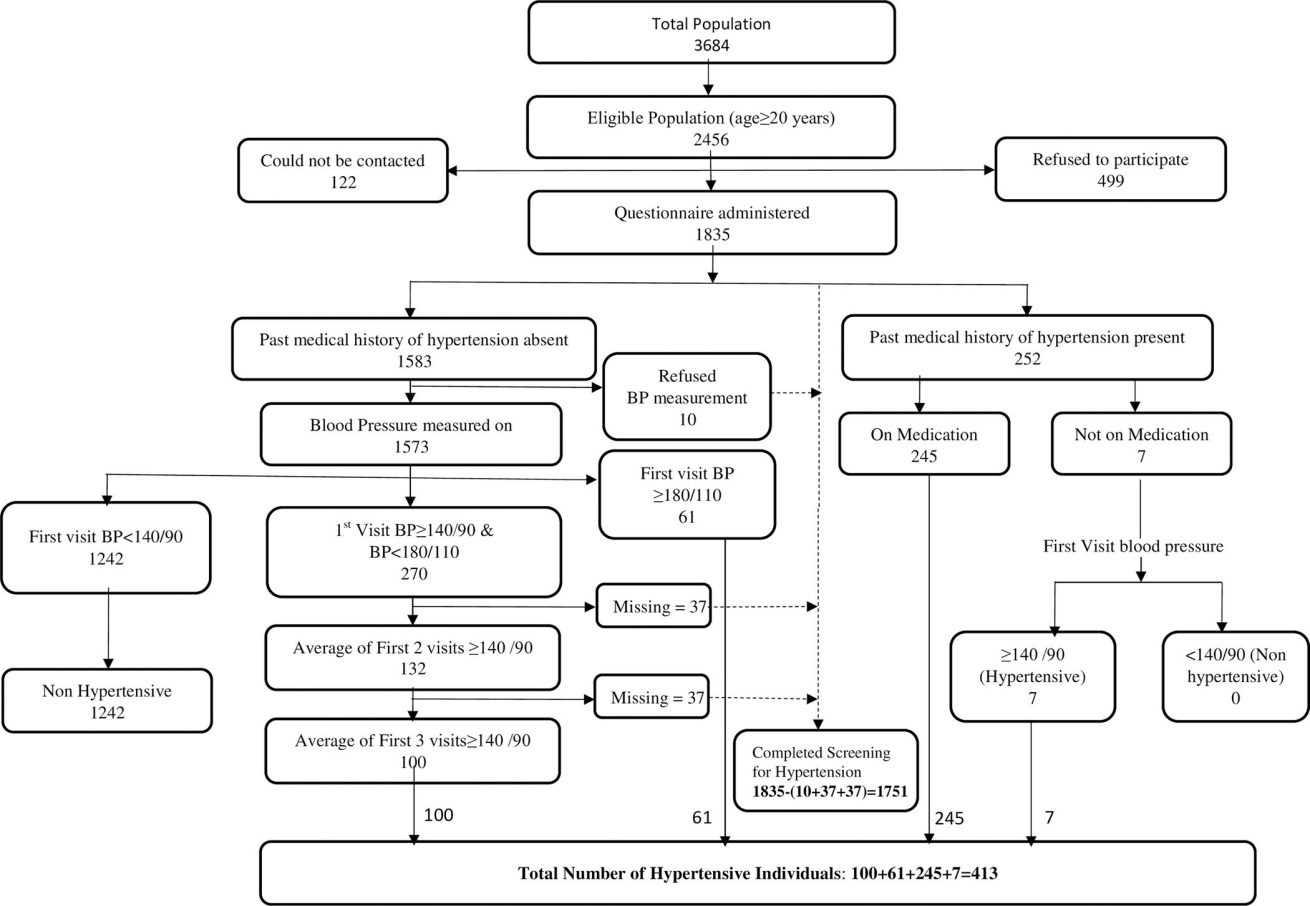
Summary of hypertension screening.

The total population in the study villages was 3684 with an eligible population (aged≥20 years) of 2456. Among the eligible individuals, 122 could not be contacted and 499 refused to participate. The demographic characteristics of the non-participants and participants were not largely different except that relatively higher proportion of males refused to participate in the study (26% males Vs 17% females). The study questionnaire was administered to the remaining 1835 (76.3%) individuals (Fig 2). The population was middle-aged (mean = 40·0 years, SD = 14.5). Nearly one third (29.8%) of the study population was uneducated. The prevalence of current smoking was 7·4%, whereas the prevalence of current alcohol use was 25·8% (Table 1).

**Table 1:** Characteristics of the study population.

Variables	Total (N = 1835)	Hypertension (N = 413)	Diabetes (N = 189)
Mean age in years (SD)	40·0(14·5)	52·4 (12·7)	52·1 (11·2)
Women %(n)	54·6(1002)	51·33(212)	52·4 (99)
Educational status %(n)			
Not available	4·0(73)	2·2(9)	2·1(4)
Uneducated	29·8 (547)	47·0(190)	43·4(82)
Primary School	14·0(257)	14·9(60)	14·8(28)
Middle School	13·1(241)	9·9(40)	12·1(23)
High School	34·4(632)	24·3(98)	22·2(42)
Graduate	4·1(75)	3·7(15)	4·7(9)
Post Graduate	0·5(10)	0·25(1)	0·54(1)
Current Smoking % (n)	7·4 (136)	10·2 (42)	7·94 (15)
Current Alcohol use % (n)	25·8 (473)	34·5(142)	32·3(61)

### Prevalence of hypertension and control of blood pressure

Blood pressure was measured in 1751 individuals (Fig 2). Prevalence of hypertension was 23·6% (95% CI 21·6%-25·6%); 252 had past medical history of hypertension (14·4%; 95% CI: 12.7%-16.0%) and 161 were newly detected (9.2%; 95% CI: 7.8%-10.5%). Among individuals with newly detected hypertension, 61 (37·9%) had first visit systolic BP (SBP) ≥180 mm Hg or diastolic BP (DBP) ≥110 mm Hg. There were 270 individuals who had no history of hypertension and with first visit BP≥140/90 mmHg but<180/110 mmHg. However, only 100 (37·0%) of them were identified as individuals with hypertension based on subsequent measurements.

Overall, 54% of the individuals with hypertension achieved BP control status based on the end of study visit measurements. Among those with a past medical history of hypertension the control rate was 59%, while it was 46% in the newly screen detected individuals with hypertension. Among those with a past medical history of hypertension the BP control rate improved from 47·0% at baseline to 59·0% at endline (McNemar’s chi square p <0.0001). Further, 31·2% of all individuals with hypertension achieved BP control status for at least 60% of the cumulative follow-up visits. Among individuals with a past medical history of hypertension at baseline, the pre-post mean differences in SBP and DBP were 6·5 mmHg and 5·9 mmHg, respectively (Table 2). Similarly, among the newly detected individuals with hypertension, the average reduction in SBP and DBP were 18·1 mm Hg and 15·3 mm Hg, respectively (Table 2).

**Table 2:** Comparison of change in blood pressure.

Category of individuals with Hypertension	Blood pressure	Baseline	Endline	p value
With past medical history of hypertension	Mean SBP in mmHg (SD)	139·7(24·6)	133·2(22·4)	<0·001
With past medical history of hypertension	Mean DBP in mmHg(SD)	88·4(16·8)	82·5(13·5)	<0·001
Newly detected with Hypertension	Mean SBP in mmHg (SD)	154·8(20·6)	136·7(18·9)	<0·001
Newly detected with Hypertension	Mean DBP in mmHg(SD)	103·1(14·2)	87·8(12·4)	<0·001

### Prevalence of diabetes and control of blood sugar

**Fig 3:**
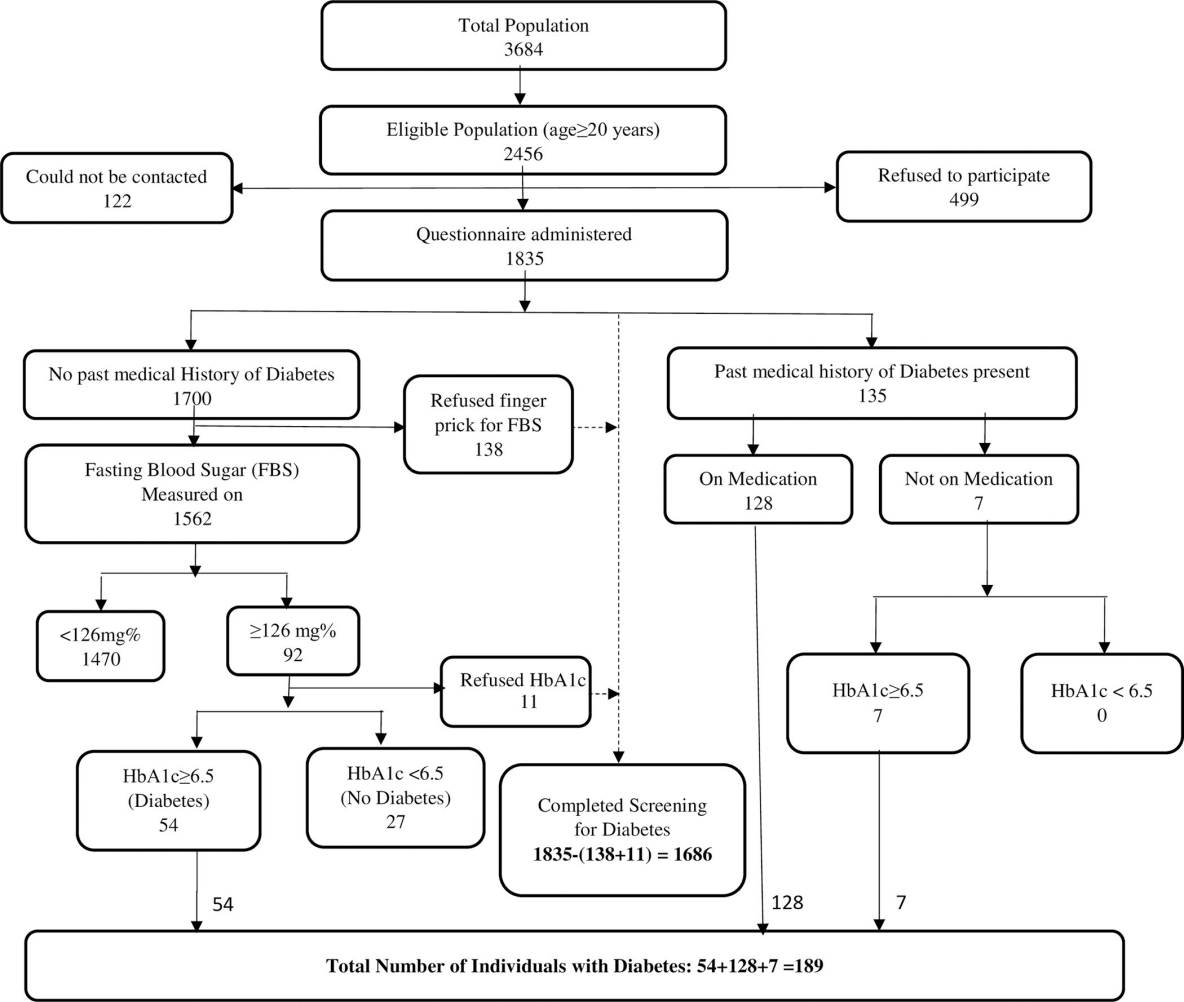
Summary of diabetes screening.

Screening for diabetes mellitus was done in 1686 individuals. The total number of individuals with diabetes was 189 (11·2%; 95% CI: 9·7% -12·7%) Past medical history of diabetes was present in 135 individuals (8.0%; 95% CI: 6.7%-9.3%); of them 128 were currently on medication and seven were not. Diabetes was confirmed in all seven of those who were not on any medications. Screening contributed to detecting diabetes in 54 additional individuals (3.2%; 95% CI: 2.4%-4.0%) (Fig 3).

Among individuals with a past medical history of diabetes, 32·0% had their blood sugar under control at baseline as measured by their HbA1c. Based on the end of study visit HbA1c measurements, blood sugar was controlled in 34·0% of all individuals with diabetes; 34·4% in those with a past medical history and 33·3% in those newly detected. Further, 15·4% of all individuals with diabetes achieved fasting blood sugar <126 mg/dl in at least 60% of the cumulative follow-up visits.

Among individuals with a past medical history of diabetes, the mean fasting blood sugar did not change much from baseline to endline (163·8 mg/dl to 170·0, p = 0·300); but the mean HbA1c decreased from 8·3% at baseline to 8·0% (p = 0·03) at endline (Table 3). Among newly detected individuals with diabetes, the mean fasting blood sugar decreased from 192·8 mg/dl at baseline to 166·3 mg/dl (p = 0·007) at endline and mean HbA1c decreased from 9·2% at baseline to 8·3% (p = 0·006) at endline (Table 3).

**Table 3:** Comparison of change in blood sugar and HbA1c levels.

Category of individuals with Diabetes	Variable	Baseline	End-line	P value
With past medical history of diabetes	Mean Fasting Blood Sugar in mg/dl(SD)	163·8(65·4)	170·0(70·5)	0·300
With past medical history of diabetes	Mean HbA1c in %(SD)	8·3 (2·2)	8·0(1·9)	0·030
Newly detected with diabetes	Mean Fasting Blood Sugar in mg/dl(SD)	192·8(65·0)	166·3(64·2)	0·007
Newly detected with diabetes	Mean HbA1c in %(SD)	9·2(2·5)	8·3(2·1)	0·006

### Performance of ASHAs

Monthly follow-up visits were duly completed in 88·8% and 90·8% of individuals diagnosed with hypertension and diabetes, respectively; and quarterly follow-up visits including Skype consultations were completed in 79% of those due for follow-up.

## Discussion

Our study demonstrates the feasibility of implementing a novel model in detection and management of hypertension and diabetes in resource poor settings. The intervention model is unique due to the following reasons; a) employs a combination of task-sharing and mHealth strategies, b) incorporates technological capabilities of integrating both screening and evaluation tools in one single mHealth application, c) facilitates direct entry of all objective measurements into the EHR, d) enables electronic consultation between the patient and the study physician that permits real time prescription of appropriate medicines for the control of BP and blood sugar. The technology enabled and trained NPHW shared the responsibility of screening, risk management, and maintenance of the electronic health records of hypertension and diabetes patients. The implementation study highlights the acceptability of the intervention model in community settings, and shows improvement in clinical outcomes in terms of BP and blood sugar control at the community level.

The relatively high participation rate in screening and follow-up demonstrate the acceptability of the intervention strategy at the community level. Identification of NPHWs from the study villages itself may have improved the acceptability of the intervention. However, the study NPHWs were above average in their capabilities, motivation and performance in the field. Additionally, the intensive in-office and long duration of on-field supervised training are quite demanding for an average ASHA. It is therefore, a challenge to maintain the same quality and standard in scale-up operations of the developed intervention strategy. Involving higher cadres of NPHW in implementation with better education and skills may help to improve the performance indicators of the intervention strategy.

The digital health application developed as part of the mHealth strategy integrated a structured framework for screening and follow-up. With the help of internet, it facilitated electronic video consultations with the study physician. In India, NPHWs are not allowed to prescribe medicines for management of hypertension and diabetes. The electronic consultation and two-way real-time access to EHR both by the physician and NPHW resolved this issue and helped us to provide on-time treatment. The timely treatment may have improved the confidence of the patients in the intervention model and resulted in better clinical outcomes. We had involved ANMs, another cadre of NPHW other than ASHAs, in drawing venous blood from the selected participants for HbA1c assessment. However, it is possible to integrate a point of care (POC) HbA1c measurement device with the mhealth application. Several POC devices are available now in the market and they often require a small sample of blood (5μl) from a finger prick for running on-site HbA1c assay. Even lay people with minimal training can run the test and the results will be available in about five minutes[12,13]. Although POC was preferable, in our environment the alternative of venous blood for HbA1c was cheaper.

About one fifth of the population in our study had hypertension, which is similar to the observed prevalence of hypertension in other studies from rural south India [5,14]. Diabetes was prevalent in about one tenth of our study population. This is a little higher than the prevalence of diabetes reported from erstwhile state of rural Andhra Pradesh where one in seventeen individuals had diabetes [6]. The higher prevalence of diabetes in our study could be due to the proximity to a peri-urban population. Comparable prevalence suggests effective detection of cases in our study.

In our study population, nearly half and one third of individuals with past medical history of hypertension and diabetes had their BP and blood sugar under control, respectively. The BP control rate in our study population at baseline is much higher than that reported in a systematic review of rural Indian data[5] Irrespective of the relatively better control rates at baseline, there was substantial improvement in control rates of BP in individuals with hypertension. Additionally, the impact of the intervention at the population level is likely to be high as it also helped to detect a large proportion of individuals with hypertension and diabetes who were otherwise not aware about their condition. Importantly, the intervention not only helped individuals with past medical history of hypertension and diabetes to achieve better BP and blood sugar control rates, but also in newly detected individuals with hypertension and diabetes. The overall reduction in mean BP in our study is relatively lower than previously published data from primary care settings[15]. However, the reductions in BP and HbA1c in our study are comparable to those observed in a recently concluded mHealth study conducted in hospital settings, wherein tablet computers enabled with software for data collection and facilitating decision making for treatment were used as part of the mHealth strategy[16].

Being a demonstration project on a small scale without a comparison group, our results have limited generalizability. Therefore, large implementation studies of our intervention strategy of combining both mHealth and task-sharing strategies are required for scale-up.

## Conclusion

Our study demonstrates the feasibility, and local acceptability of implementing a mHealth and task-sharing intervention model for hypertension and diabetes detection and control in community settings in India. Our strategy holds promise to facilitate delivery of care on a continuum for hypertension and diabetes in resource poor settings.

## Supporting information

S1 File Manuscript raw data. (XLSX)
